# A non-invasive online photoionization spectrometer for FLASH2

**DOI:** 10.1107/S1600577515022675

**Published:** 2016-01-01

**Authors:** Markus Braune, Günter Brenner, Siarhei Dziarzhytski, Pavle Juranić, Andrey Sorokin, Kai Tiedtke

**Affiliations:** a Deutsches Elektronen-Synchrotron DESY, Notkestrasse 85, 22607 Hamburg, Germany; b Paul Scherrer Institut, 5232 Villigen PSI, Switzerland

**Keywords:** free-electron laser, soft X-ray, wavelength, photoionization, rare gases

## Abstract

The stochastic nature of the self-amplified spontaneous emission (SASE) process of free-electron lasers (FELs) effects pulse-to-pulse fluctuations of the radiation properties, such as the photon energy, which are determinative for processes of photon–matter interactions. Hence, SASE FEL sources pose a great challenge for scientific investigations, since experimenters need to obtain precise real-time feedback of these properties for each individual photon bunch for interpretation of the experimental data. Furthermore, any device developed to deliver the according information should not significantly interfere with or degrade the FEL beam. Regarding the spectral properties, a device for online monitoring of FEL wavelengths has been developed for FLASH2, which is based on photoionization of gaseous targets and the measurements of the corresponding electron and ion time-of-flight spectra. This paper presents experimental studies and cross-calibration measurements demonstrating the viability of this online photoionization spectrometer.

## Abstract 2

A description of the design of an instrument for FEL wavelength monitoring based on photoionization of rare gases is given, as well as a report on calibration and characterization studies.

## 1. Introduction   

Over the past decade the free-electron laser in Hamburg (FLASH) has served as a user facility (Ayvazyan *et al.*, 2006 ▸; Ackermann *et al.*, 2007 ▸; Tiedtke *et al.*, 2009 ▸) providing highly intense short-pulsed radiation. Peak and average brilliance of FLASH exceed both modern synchrotron facilities and laser plasma sources by many orders of magnitude. The soft X-ray output possesses unprecedented flux of about 10^13^ photons pulse^−1^ with pulse durations in the femtosecond range and a high level of coherence.

Currently, a major upgrade to expand the facility and double the user capacity is under way. In the FLASH2 project a new tunnel housing a new variable-gap undulator beamline has been added to the existing facility and, in its final stage, up to six photon beamlines will be built in the new experimental hall (Ploenjes *et al.*, 2013 ▸). Both FLASH1 and FLASH2 can operate simultaneously in the soft X-ray spectral region and are based on the self-amplified spontaneous emission (SASE) process in which laser emission is built up from spontaneous undulator emission (Ackermann *et al.*, 2007 ▸).

Owing to the stochastic nature of the SASE process, individual FLASH pulses differ in their intensity, temporal structure and spectral distribution. Hence, a single-shot characterization of each single pulse is mandatory for most user experiments. This requires diagnostics tools which operate in parallel to the user experiments in a non-destructive way. At FLASH, several methods, which are based on atomic photoionization, have been developed to measure these parameters. Some of these devices have already become a standard at several free-electron laser (FEL) facilities, for example the gas monitor detector (Richter *et al.*, 2003 ▸; Tiedtke *et al.*, 2008 ▸) to measure the absolute pulse energy, which is used at FLASH and also at the LCLS (Tiedtke *et al.*, 2014 ▸; Moeller *et al.*, 2015 ▸).

In the following we will describe the online photoionization spectrometer (OPIS) which detects photoelectrons and photoions created upon the photoionization process in the interaction volume with time-of-flight spectrometers. The wavelength is determined by means of well known binding energies and partial cross-section data of the target species. Due to the absence of any optical elements and a low target gas pressure the instrument is completely non-invasive regarding the beam path and basically transparent for user experiments. Ion and electron time-of-flight spectrometers in combination with fast digitizers allow for single-bunch resolved online wavelength monitoring. Two OPIS devices will serve at FLASH2 as main monitors of the FEL wavelength.

## 2. Wavelength measurement principles   

For wavelength determination, measured signatures in the time-of-flight spectra are evaluated with respect to intensity and arrival time. The analysis is based on well known literature data of relative photoionization cross sections (Suzuki & Saito, 1992*a* ▸,*b* ▸; Suzuki & Saito, 2001 ▸) and binding energies (Kramida *et al.*, 2014 ▸; Thompson, 2009 ▸) of the target gases.

### 2.1. Ion time-of-flight spectrometer   

**Figure 1:**
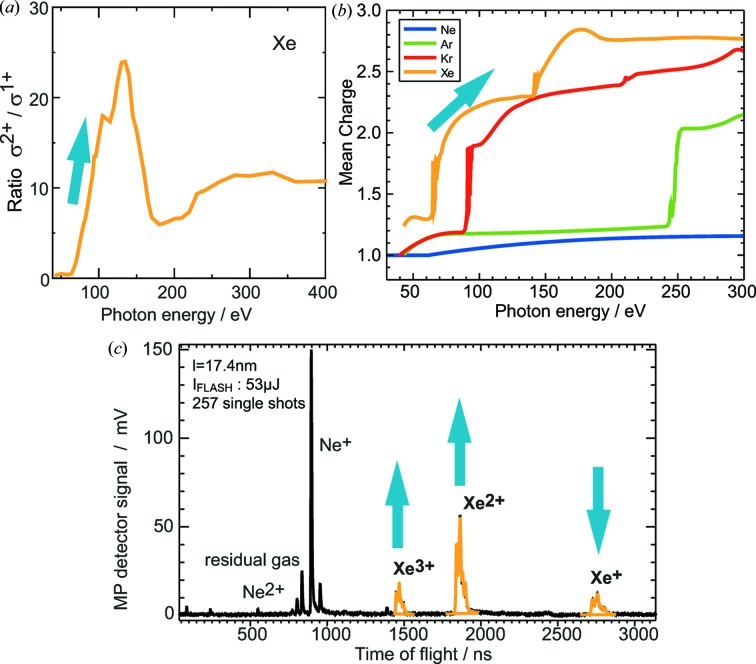
(*a*) Example plot of a partial cross-section ratio 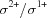 for xenon. (*b*) Reference data (Suzuki & Saito, 1992*a* ▸,*b* ▸, 2001 ▸) for the mean charge of rare gas ions after photoionization *versus* photon energy. (*c*) Example of an ion time-of-flight spectrum averaged over 257 single shots of FLASH at λ = 17.4 nm using Xe and Ne as target gases. For a decreasing FEL wavelength, the intensities of the Xe^3+^ and Xe^2+^ signals increase whereas the Xe^+^ line decreases, leading to a change in the mean charge as well as the cross section ratio values, as illustrated by the arrows.

The signals of different ionic charge states of the photoionized target atoms are located at different positions in the time-of-flight mass spectrum. Since cross-section ratios *R* [equation (1)] of these charge states and the mean charge γ [equation (2)] are explicit functions of the wavelength over wide ranges (Suzuki & Saito, 1992*a* ▸,*b* ▸, 2001 ▸), as shown exemplary in Fig. 1 ▸, we are able to deduce the wavelength from the measured normalized intensities of the different charge states 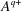 in the ion spectrum:
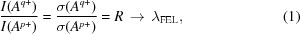


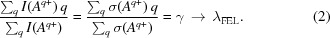
A shift of the FEL wavelength results in a change of the signal intensity ratios of the ionic charge states. The steeper the slope of the reference curve, the higher is the wavelength sensitivity. As can be seen in Fig. 1 ▸, a proper choice of the target gas for different wavelength regions is essential for an effective wavelength determination. Of course, at least two different charge states are mandatory for this method and, therefore, it works only above the double ionization threshold of the chosen target gas.

### 2.2. Electron time-of-flight spectrometer   

In the electron time-of-flight spectra, the arrival time of the photoelectrons reflects their kinetic energy which is in turn directly connected to the photon energy. The only reference numbers from the literature needed here are the binding energies of the observed electron orbitals:
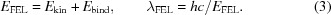
With the binding energy of 12.1 eV of the outer valence 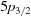 electrons of the heaviest rare gas xenon it is possible to measure the according photoline with FEL wavelengths up to 100 nm. Therefore, the full FLASH wavelength range of 60 nm to 4.2 nm can be covered. In principle, only a single photoelectron feature of any target gas would be enough to measure in the entire wavelength range above its threshold. An advantageous feature of the electron time-of-flight spectrum is the fact that it displays the full electron kinetic energy range, *i.e.* the complete wavelength range, at a time.

**Figure 2:**
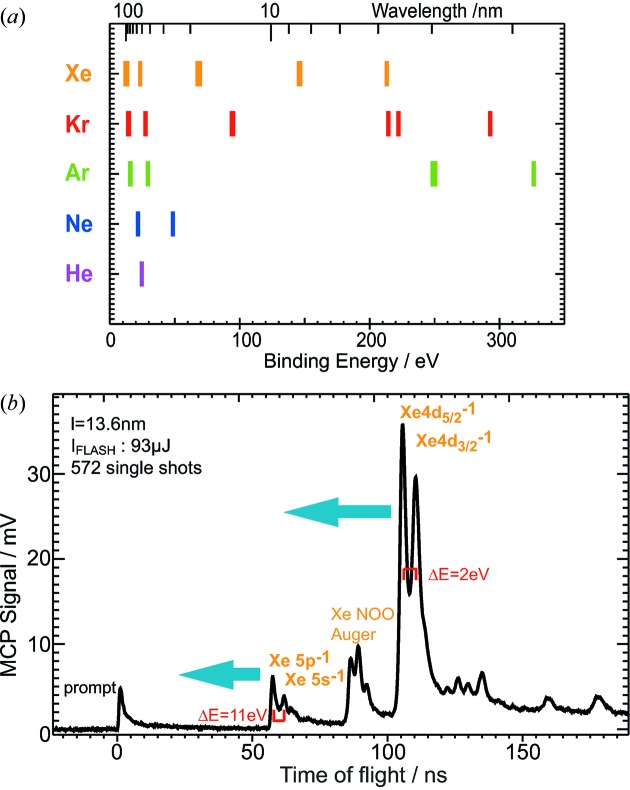
(*a*) Binding energies of the electron orbitals of the rare gases *versus* photon energy and wavelength in the FLASH wavelength range. (*b*) Example of an average electron time-of-flight spectrum from 572 single shots of FLASH at λ = 13.6 nm. For a decreasing FEL wavelength the xenon photolines shift towards smaller arrival times as illustrated by the arrows. The shift will be larger for the low kinetic energy 4*d* lines than for the faster 5*p* electrons due to the non-linear energy scale. To give an idea of the energy scale, the markers indicate the Xe 4*d* energy splitting and the difference in kinetic energy between the 5*p* and 5*s* photoelectrons.

Changing the wavelength causes a shift of the photoelectron lines in arrival time (see Fig. 2 ▸). Because of the non-linear relation between energy and time, *t* ≃ 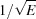, this shift is larger for slow electrons. The kinetic energy resolution and hence the wavelength sensitivity is higher for longer electron flight times. With respect to wavelength determination, one can benefit from this fact by proper selection of a target gas with convenient electronic orbitals having low kinetic energies at the FEL wavelength at hand. In addition, the photoelectrons can be decelerated by applying retardation potentials to the spectrometer in order to increase the resolution.

## 3. The OPIS device   

**Figure 3:**
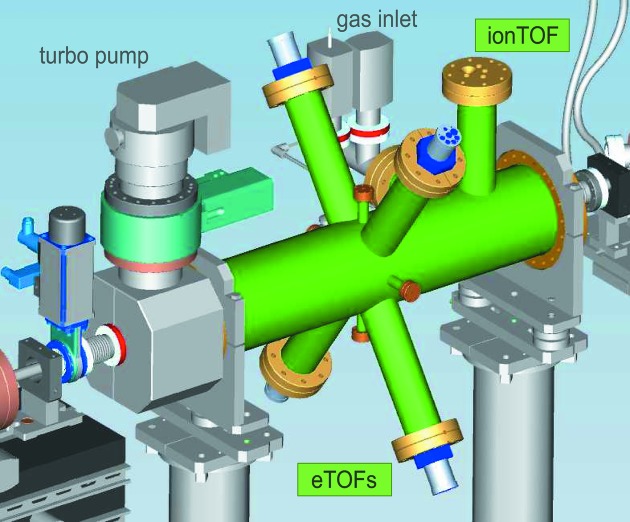
A simplified CAD model of the OPIS device. A 0.7 m-long CF 150 tube constitutes the body of the μ-metal vacuum chamber (green) of the instrument. Four CF 63 vacuum ports at angles of 54.7° with respect to the horizontal plane as well as one vertical CF 63 port contain the electron and ion time-of-flight spectrometers. Additional ports are provided for gas inlet tubes and pressure gauges. The system is pumped by a turbomolecular pump with a pumping speed of 300 l s^−1^ attached to the end of the chamber.

The OPIS device is a CF 150 tube vacuum chamber of 0.7 m in length with ports for the target gas inlet system and four electron as well as one ion time-of-flight (TOF) spectrometers (Fig. 3 ▸). Mostly rare gases are used as photoionization targets which have a purity of 99.99% or better. The target gas is introduced into the chamber in an effusive gas beam at the electron and ion spectrometer positions through apertures of 0.5 mm and 1 mm in diameter, respectively. Typical target gas pressures are in the range of 10^−7^ hPa which allows a photon transmission to the user experiment of basically 100%.

The ion-TOF spectrometer collects all photoions created by FLASH using an electric extraction field and detects them with a multiplier (ETP 14882). The homogeneous acceleration field is created between two circular extractor plates with a diameter of 54 mm at a distance of 25 mm. Potentials of typically +1700 V and −2000 V, respectively, are applied to accelerate the photoions into a 100 mm-long drift tube, in accordance with the Wiley–McLaren-condition (Wiley & McLaren, 1955 ▸). The operation voltage of the multiplier at the end of the drift tube is in the range 2600–3000 V.

Four electron TOF spectrometers detect photoelectrons within a fixed solid angle by microchannel plate (MCP) detectors. All of them are mounted at an angle of 54.7° with respect to the horizontal radiation polarization axis of FLASH. At this angle the photoelectron intensity is independent of any angular distribution effects of the photoemission process (Hemmers & Lindle, 2001 ▸; Becker & Shirley, 1996 ▸). The relatively small acceptance angle of approximately 6° is defined by a 3 mm entrance aperture being 28 mm apart from the center of the interaction region. The total distance from the interaction point to the detector surface is 309 mm. The 274 mm-long drift tube is divided into three sections for applying retardation potentials in order to decelerate the photoelectrons for higher energy resolution. The MCP detector comprises a set of three MCP plates, 33 mm in diameter, matched with respect to resistance, and mounted in a Z-stack arrangement. A typical operation voltage of the detector is 2850 V. Accurate alignment to the photon beam axis is achieved by means of a centering mounting ring holding the spectrometer tips. The central position of this ring is defined by four pins on CF 16 vacuum flanges mounted in according ports in the electron spectrometer plane. With a bore hole in the pin’s axis they also serve as vacuum feedthrough and inlet needle for the target gas. Field-free conditions are important for good electron TOF operation. To reduce external magnetic fields the chamber is made from μ-metal.

A displacement of the photon beam from the nominal centered beam position would result in a shifted photoelectron arrival time compared with the nominal arrival time in a single electron time-of-flight spectrometer. By deploying a pair of two electron spectrometers measuring photoelectrons in opposite directions, this can be distinguished from real wavelength changes since a shift due to beam displacement should have opposite signs in the two spectrometers. In principle, with two pairs of spectrometers in the OPIS instrument, vertical and horizontal photon beam position information can be deduced by analyzing differences of photoelectron feature arrival times of all four spectrometers. Efforts in beam position monitoring are at a very preliminary stage and will not be reported here.

The detector signals of all spectrometers are sampled by means of fast ADC devices (SP Devices ADQ412/ADQ108). By recording traces of full bunch trains of FLASH containing the arrival time of the photoions and photoelectrons for each single bunch, the OPIS device is multi-bunch operation capable.

For the measurements reported here, a LeCroy DSO WR625Zi oscilloscope has been used for data recording and the wavelength analysis has been carried out within the IDL programming environment on a personal computer. A unique number which designates each FLASH light pulse, the so-called bunch-ID, is provided by the FLASH machine operation system. This number has been assigned to the recorded data in order to correlate it with the data of the reference spectrometers and other diagnostic tools, such as for intensity or beam position.

**Figure 4:**
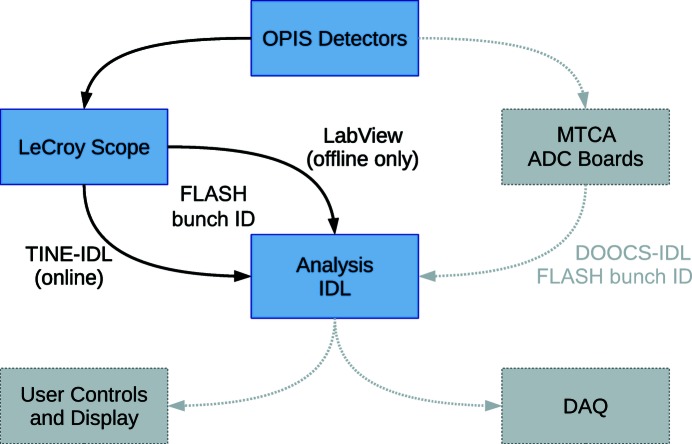
Scheme of data acquisition. In the test measurements the detector signals have been recorded by an oscilloscope and transferred together with the respective FLASH bunch identifier number to a PC by a TINE server or a LabView tool for online or port-recording analysis, respectively. At FLASH2, digitizer cards in MTCA systems will be used for recording. Measurement controls and wavelength analysis will be embedded in the FLASH control system (DOOCS), and a connection to the FLASH DAQ system will be established for data storage.

At FLASH2, MTCA systems with digitizer cards (SP devices ADQ108 and ADQ412) will be used for data recording allowing for higher data throughput (Fig. 4 ▸).

## 4. Experiments   

To evaluate the performance of the OPIS instrument, measurements have been conducted using a simplified setup with only one electron and one ion spectrometer. This test setup is located in the photon diagnostics section of the FLASH1 tunnel before any optical elements for photon beam transport. The general task was the determination of the most appropriate operation parameters in order to optimize the detector signal quality and to avoid effects which may distort the wavelength measurement results, such as for example detector saturation or space charge effects. Additionally, in a special experimental run dedicated to the resolving capabilities in wavelength determination (see §4.1), measurements have been performed with one of the new fully equipped OPIS devices set up at the user experimental station at the end of the plane-grating (PG) monochromator beamline. All results reported in the following are measured in single-bunch mode of 10 Hz operation of FLASH.

A major part of the campaign was dedicated to the wavelength calibration of the electron spectrometer. To that end, several grating spectrometers available at FLASH have been involved in the measurement for comparison and to serve as a wavelength reference. Most importantly, the PG monochromator beamline in spectrometer mode, referred to as the PG spectrometer (PGS) (Martins *et al.*, 2006 ▸; Gerasimova *et al.*, 2011 ▸), and the mobile compact spectrometer (CS) (Frasetto *et al.*, 2011 ▸) which can be installed at any experimental station of the FLASH beamlines were used for that purpose. The uncertainty of wavelength measurements of the PG spectrometer varies to some extent over the complete FLASH wavelength range. In general, the PG wavelength uncertainty can be specified to be 0.02 nm or better. The compact spectrometer served as a reference for measurements at 4, 6 and 8 nm. In this wavelength interval the uncertainty using the 2400 lines mm^−1^ grating ranges from 0.03 to 0.05 nm, respectively (Frasetto *et al.*, 2011 ▸).

Simultaneous measurements with the OPIS and grating spectrometers have been conducted at a number of different wavelength values throughout the FLASH wavelength range. In most measurements the wavelength was scanned towards shorter values by increasing the accelerator energy. During a measurement shift of 12 h, a total tuning range of about 1 nm could be covered in steps of typically 0.1 nm.

### 4.1. Cross-calibration campaign   

Before measurement of the FEL wavelength, both ion and electron spectrometers had to be calibrated. In particular, for the electron spectrometers, the determination of the conversion function assigning kinetic energy to measured time-of-flight requested a number of experimental runs at different wavelength.

Regarding the ion spectrometer, the analysis of intensities demands a calibration of the detector response depending on the input signal strength. Commonly, multipliers with typical dynode materials such as Be–Cu as well as MCP-based detectors show amplification factors *G* which depend nearly linearly on the velocity *v* of the impinging ions 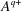 carrying a charge *q* (*e.g.* Schram *et al.*, 1966 ▸; Stockli & Fry, 1997 ▸). For the model used in the ion spectrometer and similar multipliers, this behavior could be confirmed already in earlier measurements at a synchrotron source (Juranić *et al.*, 2009 ▸), at FLASH (Guichard *et al.*, 2013 ▸) and at SACLA (Kato *et al.*, 2012 ▸). Accordingly, intensity correction factors have been introduced for the ion spectra analysis,
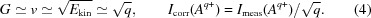
With respect to the electron spectrometer calibration, dedicated experimental runs have been used to collect the required database. In the analysis, values of kinetic energies could be attributed to the measured photoelectron time-of-flight by using equation (3) with the wavelength values measured by the reference spectrometers. With a selection of FEL wavelengths and the analysis of the various electronic orbitals of the available rare gases, a large range of electron kinetic energies could be covered.

**Figure 5:**
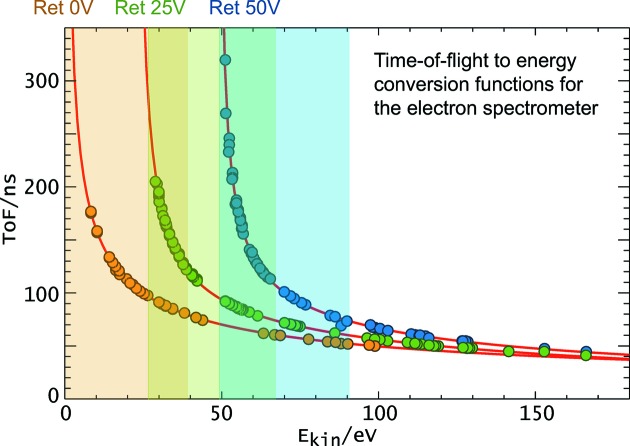
Conversion functions representing the relation between the time-of-flight and the kinetic energy of the electrons. Three different curves for retardation voltages 0 V, 25 V and 50 V are shown as an example. The curves have been calculated by means of the least-squares fit procedure using the experimental values (circles) measured with different rare gases (Ne, Ar, Kr, Xe) at different wavelength as input data. The shaded areas highlight the range of about 0 eV to 40 eV of the final kinetic energy after deceleration, which is most suited for wavelength determination. The overlapping intervals together with the variety of rare gas electron orbitals constitute a sufficient tool kit for measurements in the complete FLASH wavelength range.

With these data, time-to-energy conversion curves could be determined for a set of retardation voltages (Fig. 5 ▸). In the course of the measurements, these conversion functions could be iteratively refined until the calibration data volume was considered to be sufficiently large for precise wavelength measurements.

For wavelengths short enough to ionize lower electronic orbitals, Auger processes occur in which the re-ordering of the excited electronic state leads to ejection of electrons of a constant kinetic energy corresponding to the difference of the energy levels involved in the relaxation process. Due to the constant energy, these Auger electrons will appear at constant arrival times in the time-of-flight spectrum.

**Figure 6:**
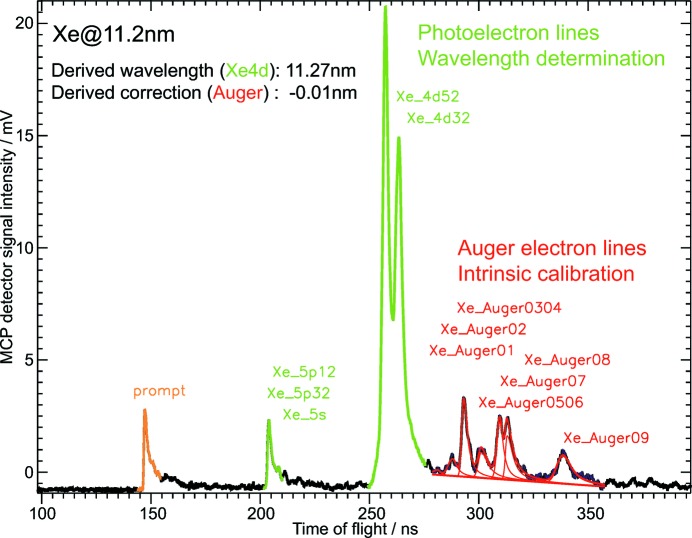
Average electron time-of-flight spectrum of 400 single shots measured with xenon at a general FLASH wavelength of 11.2 nm. A retardation voltage of 25 V was applied to the electron spectrometer. The prompt signal (orange) from fluorescence and scattered photons defines the time zero of the time-of-flight period. The photoelectron signals of different xenon orbitals (green) shift in arrival time with changing photon energy enabling FEL wavelength determination. Auger electron signals are fixed in arrival time due to their constant kinetic energy. Hence, they can serve as intrinsic calibration and for energy scale corrections when necessary. In this example, the wavelength derived by OPIS was 11.27 nm and only a minor correction of −0.01 nm has to be applied. This is in good agreement with the wavelength of 11.24 nm measured with the PG spectrometer.

Hence, in contrast to the photoelectrons which shift in arrival time according to the photon energy and serve for wavelength determination, Auger electrons represent fixed markers on the energy scale and can serve as an intrinsic calibration (Fig. 6 ▸). The time-of-flight positions together with the respective kinetic energies of various Auger lines of krypton and xenon have been used for the compilation of the calibration data set in addition to the reference spectrometer data.

The conversion functions have been derived by a least-squares fit procedure using the experimental values for time-of-flight and corresponding nominal electron kinetic energy as input data. A phenomenological approach has been used for the model function which contains up to five effective parameters 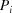,
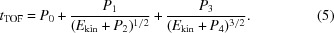



In terms of kinetic energy, the resolution of the electron spectrometers is essentially sufficient to resolve all relevant rare gas photoelectron features, such as for example the *np* valence doublets. However, the nominal resolving power 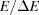 of up to 1000 can be achieved only for small interaction volumes defined by a focused photon beam with typical diameters of a few hundred micrometers.

To examine the OPIS resolution capabilities with respect to the FEL wavelength determination, a series of measurements has been conducted at the endstation for user experiments of the PG2 beamline with one of the new OPIS devices for FLASH2. The beam size at the position of the plane of the four electron spectrometers was approximately 200 µm. The FLASH wavelength was set to λ = 14 nm and average xenon spectra of about 1000 single-shot spectra have been recorded. At this wavelength the Xe 4*d* photoelectrons have a kinetic energy of around 20 eV which is well suited for wavelength determination (see Fig. 5 ▸). The PG monochromator was used to narrow the FLASH photon bandwidth of around 2 eV to some 270 meV and to scan the photon energy within the photon bandwidth in steps of about 0.05 eV. The according shift in arrival time of the position of the Xe 4*d*_5/2_ photoline in the spectra has been observed.

**Figure 7:**
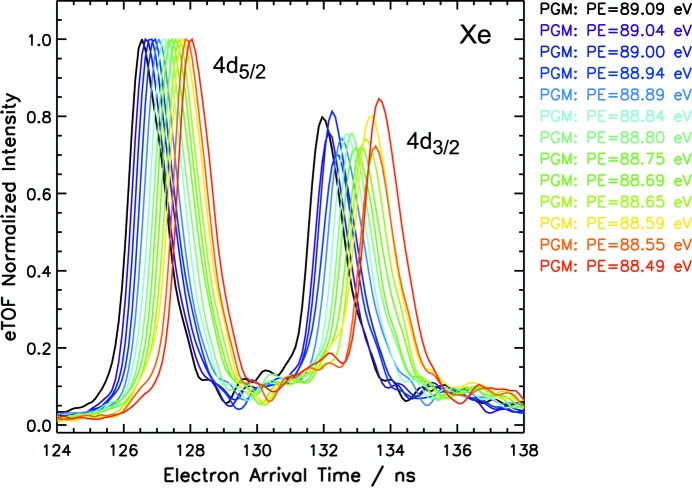
The region of the Xe 4*d* photoline in a series of electron time-of-flight average spectra of 1000 shots recorded at the PG2 beamline. The spectrum intensity has been normalized to the Xe 4*d*_5/2_ amplitude for comparison. The abscissa values specify the time elapsed after the data acquisition trigger. On this scale, the prompt signal of 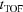 = 0 is located at 16.1 ns. With the PG monochromator in first order the photon energy was scanned between 89.09 eV and 88.49 eV in steps of about 0.05 eV. The mean of the corresponding shift in arrival time for each step is 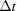 = 123 ± 15 ps. The monochromator exit slit was set to 1000 µm, corresponding to a photon energy bandwidth of 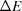 = 274 meV at 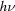 = 88.7 eV. The natural linewidth of the Xe 4*d* core-hole state is 106 meV (Masui *et al.*, 1995 ▸).

As an example, the results for one of the spectrometers are shown in Fig. 7 ▸. The scanning of the photon energy is clearly reflected in shifts of the 4*d* photoline positions by about 120 ps which are unambiguously distinguishable for two successive steps.

Therefore, the capability of OPIS to resolve small wavelength changes can be quantified to be 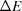 = 0.05 eV or better, in principle. On the other hand, in measurements at some location in the FLASH beamlines the full photon bandwidth of the FEL of typically 1% will affect the photoline width, and additional broadening will occur due to the fact that the photoelectrons originate from a large interaction volume of a photon beam of several millimeters in diameter. Even though only centroid positions of the photoline are decisive for wavelength determination, these broadening effects reduce the accuracy of the OPIS wavelength results.

### 4.2. Wavelength measurement results   

In the second phase of the experimental campaign, further simultaneous measurements with OPIS and grating spectrometers for arbitrary FEL wavelengths have been performed. The OPIS wavelength results derived with the established calibration have been compared with the reference spectrometer values in order to check for consistency. Again, for the most part average spectra of a few hundred FEL shots have been analyzed, which means that mean wavelength values of a few 10 s of FLASH operation have been determined.

**Figure 8:**
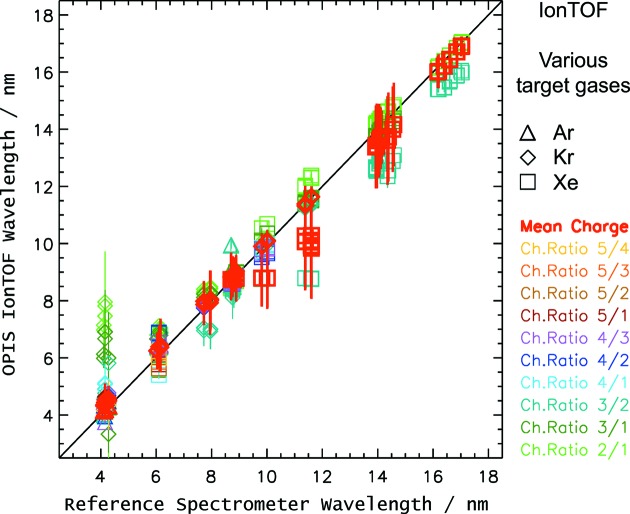
Correlation plot of wavelength results derived with the OPIS ion spectrometer and the reference spectrometers. The results are depicted with different symbols for Ar, Kr and Xe. In addition, different colors point out the various methods of wavelength analysis of the OPIS ion spectra considering different ionic charge states. Each symbol represents a mean wavelength value of a data acquisition of a few hundred FLASH pulses. The error bars indicate the wavelength value uncertainty derived from fit parameter errors.

Our data set allowed for OPIS ion spectra analysis in the wavelength range from 4 to 17 nm. The respective results for Ar, Kr and Xe are plotted against the reference values of the PG and compact spectrometers in a correlation diagram in Fig. 8 ▸. The different colors of the symbols indicate different methods of calculation in terms of the ionic charge state signals used in the wavelength determination.

The best results concerning the wavelength determination from ion signals we expected to achieve from the mean charge analysis, since it includes experimental input from all the available charge states. For our data set, according to §2.1 the most appropriate rare gas for the measured wavelengths are Ar at around 4.3 nm, Kr for 6–12 nm and Xe for 14 and 17 nm in terms of the mean charge method. In fact, considering the results of this selection the agreement of the OPIS with the reference spectrometers is of the order of 0.2 nm or better, except for Xe at 14 nm, where the deviation is of the order of 0.5 nm. As expected, measurements with Ar at wavelengths in the range 14–6 nm as well as with Xe at 6 nm and above resulted in large deviations due to the very shallow mean charge curve in this region. Those measurements have been omitted in Fig. 8 ▸. Compared with the majority of the mean charge results for Xe and Kr, there is a notably large deviation for Xe for 11.5 and 10 nm. Whereas the fact that the wavelength change by the small FEL tuning steps in the two data subsets is not correctly reflected in the results can be explained by the small Xe mean charge curve gradient in this region, the basic OPIS wavelength value is off by more than 1 nm in addition. Comparison with the according Kr results which show good agreement suggests saturation effects of the ion detection multiplier as a possible explanation, since the Xe ion signal amplitudes are two to three times larger than the Kr signals at these particular wavelengths near the xenon giant resonance.

With the evaluation of the intensity ratio of two ionic charge states, in some cases the results are in much better agreement with the reference results than with the mean charge method. For example, the intensity ratios Xe^2+^/Xe^1+^ for data points around 16.6 nm, Xe^3+^/Xe^1+^ at 11.5 nm, and Xe^4+^/Xe^1+^ at 10.0 nm give results for which the agreement is of the order of 0.1 nm or better. This is also true for krypton ratios Kr^4+^/Kr^2+^ around 7.8 nm and Kr^3+^/Kr^2+^ around 6.0 nm. Overall, however, the results for the charge state ratio method differ substantially in terms of agreement with the reference spectrometer values. Unfavorably, due to similar amplitudes and a small gradient, the ratio analysis of the most intense charge state signals for a certain wavelength does not give automatically the most reliable OPIS wavelength results, as can be clearly seen in Fig. 8 ▸, for example, for the ratio Xe^3+^/Xe^2+^.

After all, the ion results presented here would suggest an OPIS operation using a table containing mandated target gases and the most suited wavelength determination methods for all FEL set wavelengths, similar to the operation of the FLASH pulse intensity gas monitor device (Richter *et al.*, 2003 ▸; Tiedtke *et al.*, 2008 ▸). From a pragmatic perspective, this is acceptable for the purpose of having a wavelength-monitoring tool. However, the inconsistency in results of the various methods is surely not satisfying. Possible reasons for the differences are a broadband synchrotron radiation background, created by a strong bending magnet near our test setup in the FLASH1 tunnel, and saturation effects of the multiplier at high signal strengths. In addition, the approach of a general intensity correction may have to be refined to an efficiency correction depending on the charge of the ionic final state. A careful examination of these issues holds a promising perspective for improvements. Furthermore, with the convenient wavelength tuning capabilities of FLASH2 due to variable-gap undulators, effective reference data for the ionic charge state branching ratios and mean charge could be established by repeated measurements.

**Figure 9:**
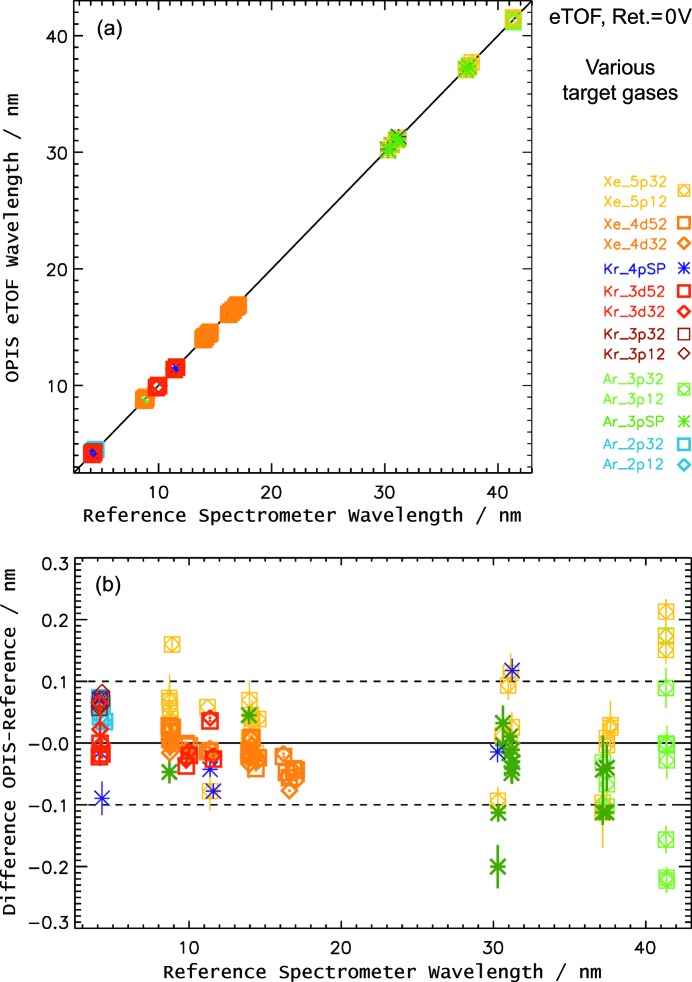
Comparison of wavelength results derived from the OPIS electron spectrometer (0 V retardation voltage) with the reference spectrometer in a correlation plot (*a*) and a deviation plot (*b*). Results for the various photoionized electron orbitals are given in different colors. The data points of the photoelectrons which are considered most suitable for wavelength determination for the respective wavelength are depicted in bold symbols. The wavelength values have been corrected according to the results of an Auger electron analysis where applicable. Each symbol represents a mean wavelength value of a data acquisition of a few hundred FLASH pulses. The error bars indicate the wavelength value uncertainty derived from fit parameter errors.

The results of the OPIS electron spectrometer are shown in Fig. 9 ▸. Measurements have been carried out at wavelengths from 4 to 40 nm and thus cover a large part of the FLASH wavelength range. For each wavelength, a particular rare gas target showing a prominent photoelectron feature at a low or at least moderate kinetic energy was chosen for wavelength determination. Nevertheless, in most cases several rare gas species have been used for a certain wavelength and, in general, all photoelectron features present in the electron spectra have been analyzed for comparison.

In Fig. 9 ▸ the results for various photolines are depicted with symbols of different colors. The data points resulting from the photoelectrons selected to be most suited for wavelength determination are highlighted with bold symbols. In two panels the derived wavelength values are compared with the corresponding reference spectrometer results in a correlation plot as well as a diagram showing the deviation between OPIS and reference spectrometers in more detail.

For wavelengths up to 30 nm, the OPIS data determined from the selected photolines generally agree with the reference spectrometer values very well within 0.1 nm or better. The other photoelectron features show a somewhat larger deviation and scattering, mostly due to lower energy resolution of the spectrometer at higher kinetic energies and lower electron signal intensity. The pronounced variance in the results at 40 nm can be explained by the increased photon beam size for long FLASH wavelengths, which is of the order of 10 mm at 40 nm, and to some extent unstable operation conditions during the experimental shift. Nevertheless, considering the FLASH photon energy, the deviation for the measurements at 40 nm in absolute terms is still within ±0.2 eV, which is comparable with the measurements at shorter wavelength.

**Figure 10:**
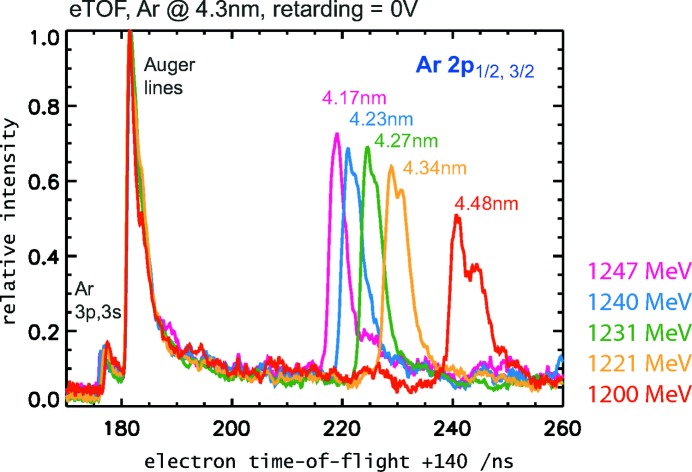
OPIS wavelength measurements to evaluate shortest FLASH wavelength values corresponding to highest accelerator energies. The argon 2*p*_3/2,1/2_ electrons with binding energies of 248.4 eV and 250.6 eV, respectively, are most suited for wavelength determination in this range. The mean kinetic energy of the 2*p* doublet increases from 27.3 eV to 47.8 eV shifting the wavelength from 4.48 nm to 4.17 nm. For each spectrum the accelerator electron energy set for FEL operation at the particular wavelength is specified in the legend in the corresponding color. The measurements have been conducted in one experimental run of 12 h.

Fig. 10 ▸ illustrates measurements in the low-wavelength region 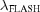 < 4.5 nm at the highest electron accelerator energies of FLASH. In this case the mobile compact spectrometer is the most suited device served as a reference grating spectrometer, since it was designed to cover short wavelengths in order to measure the higher harmonics of the FEL radiation together with the fundamental. With the calibration already at hand and using the argon 2*p* photoelectrons for wavelength determination, the OPIS measurements contributed to confirm the appropriate operation parameter set of the compact spectrometer and to determine the value of the shortest wavelength λ = 4.17 nm at the highest accelerator energy of 1247 MeV. Furthermore, the OPIS measurements have been used to tune FLASH to match the wavelength λ = 4.30 ± 0.05 nm of a narrow-bandwidth multi-layer mirror deployed in a user experiment.

As already pointed out, due to the larger photon beam the resolution of the features in the spectra is not as high as in the measurements at the monochromator PG2 beamline described in the previous chapter. This can be seen for example by comparing the xenon spectra of Fig. 7 ▸ with the argon spectrum at 4.48 nm shown in Fig. 10 ▸. There is a significant difference in the shape of the Xe 4*d* and Ar 2*p* photolines, despite the fact that the fine-structure splitting and the kinetic energy of the according photoelectrons are quite similar in both cases [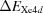 = 2.0 eV, 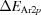 = 2.2 eV, 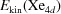 ≃ 20 eV, 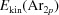 ≃ 27 eV].

Other issues regarding the conditions of the test location in the FLASH tunnel most probably affect the wavelength measurement accuracy as well, such as changing photon beam position and residual magnetic fields due to strong bending magnets close to the setup. Furthermore, these conditions depend on the FEL wavelength and may change from day to day. Since the complete data set has been accumulated in many shifts during a longer period of time, the conditions in the measurements have been partly different from those during the calibration campaign. The results of examination of Auger electron line positions to determine necessary corrections of the electron kinetic energy are a clear indication in this respect. The resulting correction values for spectra from different experimental shifts show a considerable spreading and range up to 1 eV.

Under the circumstances, which have been not optimal for photoelectron spectroscopy, the test measurements can be considered to be very successful. With the given conditions and using a moderately demanding setup in combination with a simple automatized line-fitting algorithm, it is possible to measure the FEL wavelength with an accuracy adequate to the objective to develop a diagnostic tool rather than a high-precision experiment involving a more elaborate analysis.

### 4.3. Information about the FEL spectral distribution   

The spectral distribution of the FLASH radiation should be reflected in the photoelectron line shape. To extract information from the measurements the electron time-of-flight spectra have to be converted into spectra *versus* kinetic energy using the conversion functions determined in the calibration procedure. As a fundamental task, the wavelength bandwidth of the FEL radiation can be investigated and compared with the spectral bandwidth measured with the PG spectrometer. For the spectrometer mode of the PG monochromator beamline, a Ce:YAG screen is moved into the exit slit position of the SX700 monochromator. Images of the dispersed beam pattern recorded by a CCD camera are integrated along the direction perpendicular to the dispersion axis to obtain the spectrum. Since the PG beamline alignment for this spectrometer mode does not aim for highest resolution and because of possible broadening effects due to over-exposure of the Ce:YAG screen, the measured FEL bandwidth may be regarded as an upper limit.

**Figure 11:**
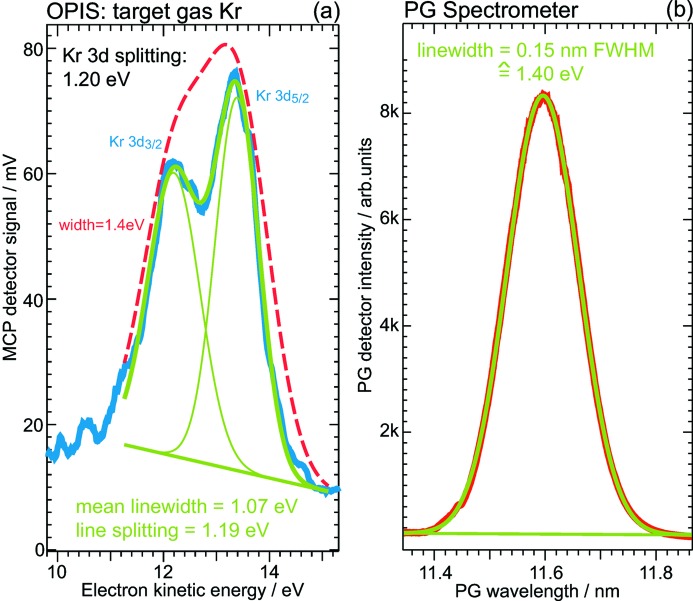
Example of a measurement of the spectral distribution of FLASH by means of an OPIS spectrum converted from time-of-flight to kinetic energy scale. (*a*) OPIS energy spectrum of Kr 3*d* photoelectrons with a spin–orbit splitting of 1.2 eV. The least-squares fit of two Gaussian profiles gives a mean linewidth of 1.1 eV (FWHM). (*b*) Corresponding PG spectrometer average spectrum. A Gaussian fit shows a FEL bandwidth of 0.15 nm, corresponding to 1.4 eV. The natural linewidth of the Kr 3*d* core-hole state is 101 meV (Hikosaka *et al.*, 2000 ▸).

A bandwidth analysis of our measurements showed a variation of the deviation of PG and OPIS bandwidth results within several 10%, with an agreement of about 20% on average. For about two-thirds of the analyzed acquisitions the OPIS bandwidth values are larger than the PG results. In a few cases the electron spectrum could reveal that the bandwidth was obviously smaller than measured with the PG spectrometer. Such an example is given in Fig. 11 ▸ depicting average spectra of about 400 single FEL shots from a simultaneous OPIS and PG spectrometer measurement at λ = 11.6 nm. The spectral distribution measured with the PG spectrometer is depicted in the right-hand panel which exhibits an almost perfect Gaussian shape indicating a decent FEL operation. A Gaussian profile fit gives a wavelength bandwidth of 0.15 nm (FWHM) corresponding to a photon energy bandwidth of 1.4 eV. The left-hand panel shows a part of the OPIS electron energy spectrum containing the krypton 3*d* photoelectron in the kinetic energy region between 11 and 15 eV. According to the PG results, the two components of the krypton doublet structure Kr 3*d*_5/2,3/2_ have been modeled by Gaussian profiles as well. The least-squares fit reproduces the literature value for the Kr 3*d* spin–orbit splitting of 1.2 eV very well, which is proof that the conversion procedure gives the correct energy scale. The mean linewidth of the two components of the Kr 3*d* doublet is determined to be 1.1 eV which is smaller compared with the PG spectrometer result by 24%. However, this difference is significant, because with a photon bandwidth of 1.4 eV, as measured with the PG spectrometer, one should not be able to resolve the Kr 3*d* fine structure, as illustrated by the dashed red line in Fig. 11(*a*) ▸. This particular example demonstrates the informative value of photoelectron spectra analysis.

The natural width of photolines, relevant for photoionized states which can decay *via* Auger processes, are of the order of 100 meV (Masui *et al.*, 1995 ▸; Hikosaka *et al.*, 2000 ▸; Carrol *et al.*, 2001 ▸) and should play no role in a spectral distribution analysis. From a general point of view, however, signals of non-decaying singlet electron orbitals like He 1*s* are better suited for studies of this kind. This applies especially when the FEL operation conditions are not as favorable as in the example shown above, but the spectral distribution exhibits asymmetric shape or side maxima.

### 4.4. Towards single-shot wavelength monitoring   

For a reliable wavelength determination from single-shot electron spectra the data quality of the photoelectron features in the recorded traces has to be sufficient. The number of photoelectrons originating from a certain electron orbital has to be sufficiently large to form a photoelectron signature predominant over stray electron peaks and high-frequency signal noise. Of course, this is achieved more easily for high FEL photon pulse energies. Furthermore, the conditions are more favorable for measurements at longer wavelength 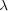 ≥ 30 nm taking benefit from high photoionization cross sections. Apart from that, one can increase the photoelectron signal strength just by increasing the target gas pressure. However, in our test measurement setup we were limited in that respect due to the lack of efficient differential pumping stages confining the gas load to the OPIS vacuum chamber and preventing the adjacent FLASH tunnel beamline sections from exceeding the maximum pressure limit allowed for safe beam operation. In our test measurements the heavier rare gas species krypton and xenon tend to show more random noise spikes in the single-shot spectra, most probably due to a larger number of secondary electrons compared with neon and argon. Generally, we achieved best single-shot spectra using neon with pressure settings which, however, could not be operated for a longer period of time because of the mentioned vacuum interlock reasons.

**Figure 12:**
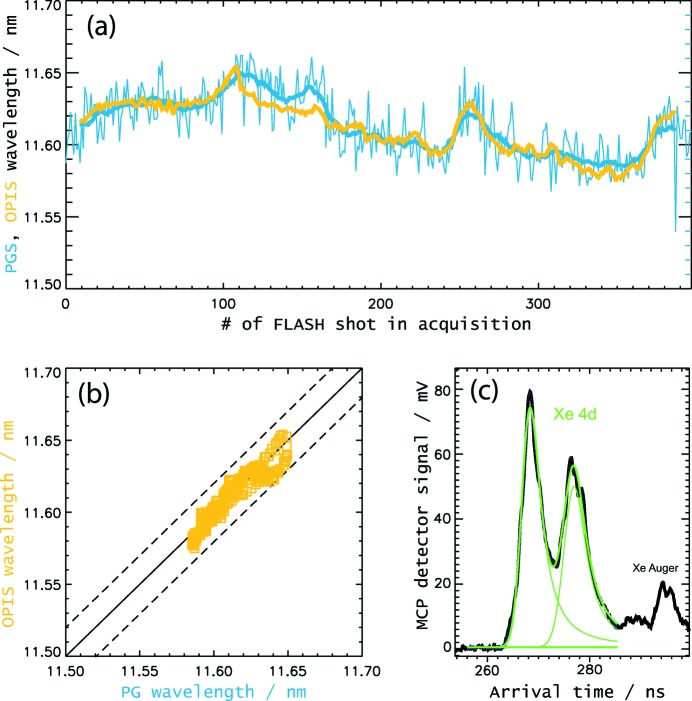
Comparison of OPIS and PG spectrometer results of a wavelength-monitoring measurement at a set value of 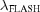 = 11.6 nm. (*a*) Wavelength values of a series of 400 FLASH pulses are shown, which, due to the FLASH repetition rate of 10 Hz, corresponds to 40 s of FEL operation. For each light pulse the average wavelength value of an interval of the last 20 shots has been determined. In the plot this average value has been attributed to the mid-shot of the 20-shot interval, *i.e.* the 11th shot. Thick yellow curve: OPIS moving average values derived from the Xe 4*d* signal of a 20-shot average spectrum. Thick blue curve: PGS moving average of 20 wavelength values derived from single-shot spectra. Thin blue curve: PGS single-shot wavelength values. (*b*) OPIS *versus* PGS results shown in a correlation plot. According to an analysis of Xe Auger electron lines, the OPIS wavelength scale had to be corrected by 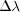 = −0.028 nm. (*c*) Example of a 20-shot average spectrum for shots #228 to #247, zoomed to the Xe 4*d* photoelectron doublet. The green curve shows the profile fit of the wavelength determination procedure.

Hence, for a test of the wavelength-monitoring capabilities of OPIS, we had to compromise in this respect by averaging over at least some ten FEL shots for a reliable wavelength determination. Fig. 12 ▸ illustrates an according wavelength-monitoring scheme with the 10 Hz repetition rate of FLASH. In a measurement with xenon at 11.6 nm, for each FLASH trigger event the actual wavelength has been derived from a 20-shot average photoelectron spectrum. Again, the wavelength was measured with the PG spectrometer at the same time. In Fig. 12(*a*) ▸ the curves of moving average values for each FLASH shot of an interval of 400 shots in total are plotted for OPIS and PGS. The OPIS average curve follows the PG curve quite closely. In addition, it reproduces fast fluctuation features which happen within a 20-shot interval, as for example in the range of acquisitions #240 to #260. This shows that this average method can provide some information about shot-to-shot wavelength changes, even if real single-shot analysis would give no accurate results. The OPIS average wavelength values have been corrected by 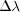 = −0.028 nm according to an analysis of a number of Xe Auger lines in the spectra. With that, the overall agreement between OPIS and PG results is of the order of 0.01–0.02 nm in this example, as can be seen more clearly in the correlation plot in Fig. 12(*b*) ▸.

## 5. Conclusion and outlook   

We showed that photoionization spectroscopy is an ideal tool for online monitoring of FEL wavelengths. It is non-invasive, covers the full spectral range of FLASH, and is well suited for single-shot resolved online wavelength monitoring during user operation. We designed an according device comprising electron and ion time-of-flight spectrometers using rare gases as target species. A calibration campaign using grating spectrometers as a reference and the first test measurements show that wavelength determination works very well with respect to the electron spectrometers. With the method of average electron time-of-flight spectra, the wavelength can be measured with an accuracy of better than 0.1 nm. We have been able to demonstrate that the bandwidth of the FLASH radiation can be determined by means of energy-converted photoelectron spectra. The results of the online monitoring tests are very promising and will certainly be improved at FLASH2 where the target gas pressure limitations of the current test setup have been overcome by proper differential pumping schemes. Accordingly designed vacuum pumping stages have been installed for two OPIS devices at the FLASH2 facility allowing for sufficiently high target gas pressures of up to 10^−6^ hPa. Regarding the results derived from the ion time-of-flight spectrometer, the measurements show that the combination of ion fragment cross-section reference data and a general multiplier detector intensity correction is not sufficient for a satisfactory accuracy of the wavelength measurement. A more careful re-analysis of the present data and confirmation measurements are necessary in order to establish an intensity calibration and a new reference data set for ion charge branching ratios. Still, the high single-shot signal intensity of the ion spectrometer offers good prospects regarding single-shot wavelength analysis. For future routine operation of two OPIS devices for wavelength determination at FLASH2 the data recording, processing and storage have to be adapted for large data volumes. The analysis routines have to be transferred to the operation environment, and computer panels for handling by non-experts have to be developed. As an outlook and to further exploit the possibilities of photoionization spectroscopy, the capabilities of photon beam position determination and monitoring shall be investigated. A preliminary analysis of data of the two pairs of electron time-of-flight spectrometers mounted opposite to each other from experiments using the small beam at the FLASH PG beamline endstation showed promising results in that respect. In FEL facilities with variable photon polarization, this property can also be monitored by electron time-of-flight spectrometry methods. Very recently, this has been successfully demonstrated using a set of electron spectrometers mounted at various angles in first measurements of this kind at FERMI and LCLS (Allaria *et al.*, 2014 ▸).
